# Smartphones as Sleep Duration Sensors: Validation of the iSenseSleep Algorithm

**DOI:** 10.2196/11930

**Published:** 2019-05-21

**Authors:** Matteo Ciman, Katarzyna Wac

**Affiliations:** 1 Quality of Life Technologies Lab University of Geneva Carouge Switzerland

**Keywords:** mobile phone use, mobile health, behavioral research, well being

## Abstract

**Background:**

Smartphones are becoming increasingly ubiquitous every day; they are becoming more assimilated into our everyday life, being the last thing used before going to sleep and the first one after waking up. This strong correlation between our lifestyle choices and smartphone interaction patterns enables us to use them as sensors for sleep duration assessment to understand individuals’ lifestyle and sleep patterns.

**Objectives:**

The objective of this study was to estimate sleep duration based on the analysis of the users’ ON-OFF interaction with their smartphone alone using the iSenseSleep algorithm.

**Methods:**

We used smartwatch sleep assessment data as the ground truth. Results were acquired with 14 different subjects collecting smartwatch and smartphone interaction data for up to 6 months each.

**Results:**

Results showed that based on the smartphone ON-OFF patterns, individual’s sleep duration can be estimated with an average error of 7% (24/343) [SD 4% (17/343)] min of the total duration), enabling an estimate of sleep start and wake-up times as well as sleep deprivation patterns.

**Conclusions:**

It is possible to estimate sleep duration patterns using only data related to smartphone screen interaction.

## Introduction

In the last decade, smartphones and mobile or connected devices, in general, are taking on a bigger role in our everyday life. Day by day, the time humans spend interacting with their smartphone is increasing [1], both because these devices can now serve more tasks, thereby helping us along daily life activities (eg, for navigation and communication) and also because they are designed to be engaging. Moreover, smartphones are equipped with several built-in sensors, for example, accelerometer, light sensor, and microphone that can provide valuable data, which can be used to get an insight into an individual’s life. For example, smartphones can be instrumented to provide recommendations about lifestyle and to follow a specific exercise regimen [2], understand the user’s stress level [3], or just provide overall support for lifestyle choices (eg, for exercise and nutrition) to facilitate better aging [4].

One of the most important aspects of everyday life of individuals that has gained a lot of attention recently is *sleep*. People’s feelings and actions throughout the day are strongly correlated with how much they slept (ie, the sleep duration) and how well they slept (ie, the sleep quality). In general, sleep affects personal health. An insufficient amount of sleep can cause fatigue and lack of concentration during the day [5]. Moreover, clinical studies show that poor sleep habits and sleep disorders are related to many serious diseases, including obesity and depression [6-8]. Increasing and converging evidence indicates that much like the majority of other organisms on the planet [9], biochemistry of the human body varies predictably throughout the day [10], a phenomenon known as the circadian rhythm. It has even been proven that circadian rhythms affect our mood, levels of concentration, digestion, sleep patterns, and much more [11].

The importance of sleep in everyday life has driven researchers to study if, and to which extent, more accessible devices, for example, smartphones or wearable devices, can be used to assess sleep quantity and quality of individuals and, in the long term, help them understand how their sleep-related behaviors could be changed. For these reasons, several developments related to smart, personal, miniaturized, and affordable devices, including smartwatches such as Fitbit [12], Withings [13], and Apple Watch [14] that use an array of embedded accelerometers, temperature, and heart rate sensors to estimate the sleep of the user, will make accurate sleep information, measured at the user’s home, available to medical experts.

Moreover, a recent study by the Pew Internet and the American Life Project found that 65% of mobile phone owners (and impressively 90% of teens) sleep with their phone on or near their bed, with many users using their smartphone as an alarm clock [15]. Results presented in this study show how the closeness of the smartphone in everyday life makes this device suitable for understanding the sleep habits of its users. Compared with approaches that require external devices, by only using the smartphone, the cost of entry for a sleep analysis tool is reduced, thus making this information more accessible to everyone.

In this study, we hypothesized that it is possible by using only a smartphone, and in particular, the information related to the users’ interactions with the smartphone screen, to understand and estimate their sleep habits. In particular, we compare the sleep duration derived from the smartphone interaction patterns with the sleep duration estimated by a smartwatch worn by the healthy study volunteers during the entire day and night. In our research, we leverage the BASIS Peak Smartwatch (by Intel Corp, Santa Clara, CA, USA) [16]. In this study, we show that it is possible to estimate sleep duration of each user, based solely on the smartphone interaction datasets, collected longitudinally in a minimally obtrusive and lightweight way. On the one hand, this information can be very useful as a contextual background to better diagnose sleep-related disorders once the individual conducts the sleep lab study. On the other hand, according to the literature [17], this information can be very useful to assess (and potentially mitigate) the risk of developing an illness in the long term (eg, cardiovascular disease and diabetes) correlated with unhealthy sleep patterns. The proposed approach is not intended to facilitate real-time sleep detection and real-time intervention but to facilitate longitudinal assessment and behavior change.

## Methods

### Study Design

The goal of our research is to understand how and to what extent it is possible to use only smartphones, and in particular, the data related to the interactions of the user with the smartphone screen, to evaluate the sleeping patterns of the user and have an insight about his or her routines (eg, sleep deprivation) that could lead to a disease later in the individuals’ life.

### Sleep Logs

The first point necessary to address is related to the ground truth data, for example, sleep logs of each individual that will later be compared with the results of the smartphone-based computational modeling of the sleep behavior. The first option for annotating data was the usage of a diary where users could annotate sleep moments before going to sleep and when waking up [18,19] or daily reconstruction methods [20] to interview the users with the aim of reconstructing their daily habits, including sleep. These methods are prone to possible subjective errors and imprecision stemming from forgetfulness of the participants to complete the logs, memory bias, or providing information about when they went to bed but not necessarily when they started sleeping. For these reasons, we decided to base our research on more reliable data, which could give a precise insight into their sleeping behavior.

We provided the users with a BASIS Peak Smartwatch [16] to wear in their daily life. The Basis Health Tracker (by Intel Corp, Santa Clara, CA, USA) is a wristwatch with an embedded actigraph. Besides the standard features provided by this smartwatch, for example, step counter, calories burned, and heart rate, the important aspect of this device is that it can automatically detect sleep episodes. Given the evidence from the medical literature, BASIS can be considered as a baseline, as it is the closest to the ground truth of sleep assessment than any other self-reported method. The BASIS sleep duration estimation has been successfully evaluated within diverse studies against gold standard polysomnography, for example, by Patel et al [21], who showed no statistically significantly different results for the total sleep time comparing the 2 methods for 40 participants.

The on-board algorithm of the smartwatch provides information about the different sleep phases (rapid eye movement, light, and deep sleep) based on the user’s physiological measures; however, we are interested mainly in the following: the start, the end, and the duration of the sleep of each user. Another feature of the BASIS is the ability to identify sleep interruptions during the night. *Interrupted sleep* occurs when a subject wakes up for few minutes and, for example, goes to a bathroom, and then goes back to sleep in less than 15 min. In this case, the sleep duration for the night is reduced by the duration of the interruptions. There is also an option for *unknown values* being provided by the BASIS, which represent the cases in which the BASIS’ on-board algorithm is unable to interpret the physiological measures and to classify the type of sleep (typically such a lack of knowledge is due to insufficient heart rate coverage). In such a case, if the set of *unknown values* is between 2 sleep episodes and is less than 15 min, it is also considered as a sleep period; it is discarded otherwise.

### Smartphone Logs

This study aims to understand if it is possible to analyze the users’ interaction with the smartphone to infer their sleep patterns. For this reason, we were interested in collecting data about the interaction of the user with his or her smartphone, and, in particular, when the user turns ON and OFF the smartphone screen—assuming that it is the smartphone owner turning ON or OFF the phone; interacting with his or her smartphone and hence, not sleeping.

To collect these data, we instrumented the smartphone with the *mobile Quality of Life* logger (mQoL-log) [22]. This app, developed by the Quality Of Life Technologies (QoL) Group at the University of Geneva (Switzerland, qol.unige.ch) and currently used in the QoL Living Lab (mqol.unige.ch), can collect and register most of the events that take place in the smartphone. For example, it collects time-stamped data about the apps used (eg, Facebook and email), the screen events such as the screen being turned ON or OFF, the physical activity of the users (walking and running) and used network 3G/4G or Wi-Fi performance information. All the data collection and the task of uploading it to the dedicated QoL lab server are made automatically in the app background, and there is no interference with the daily routine of the smartphone use. In this way, the mQoL-log collects data unobtrusively, without affecting the daily life of the users. Despite all the information available and the vast data being collected from the smartphone users, the algorithm presented in this study uses only information related to the *state* of the screen (screen ON and screen OFF), to minimize the amount of data being used and its potential privacy-obtrusiveness, and hence, maximize the user acceptance for the algorithm. In particular, the mQoL-log logs 3 different events: SCREEN ON, SCREEN OFF, and screen PRESENT. SCREEN ON represents the screen being turned ON, PRESENT when the screen is unlocked and the smartphone is ready for interaction with apps, and SCREEN OFF when the screen is turned OFF. It is important to note that the ON state of screen considered in this study only corresponds to user-interaction events, that is, SCREEN ON is recorded only when the user touches the ON button and not when the screen lights up in response to, for example, notifications and without necessarily being initiated by the user.

**Figure 1:**
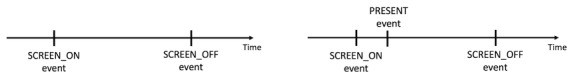
Different screen events recorded by the mQoL-log (Left: Screen ON and OFF; Right: Screen turned ON, unlocked (PRESENT), and turned OFF). mQoL-log: mobile Quality of Life logger.

Figure 1 represents the timeline of the events when the screen is turned ON and OFF, or turned ON, unlocked (PRESENT), and turned OFF.

The basic requirement of the algorithm, denoted iSenseSleep, is to identify when the user is sleeping by only analyzing the user’s smartphone interaction data, and, in particular, when the screen is turned ON and OFF.

iSenseSleep relies on two consecutive functions: (1) identifying what are all the possible time intervals along 2 consecutive days that could identify as sleep episodes and (2) evaluating these episodes to determine which one is the most probable one to represent the longest (likely overnight) sleep.

The first function works as follows: We denote the *screen event* as a tuple of SCREEN ON and SCREEN OFF events recorded consecutively by the mQoL-log (so, both cases in Figure 1 represent a screen event). Given a list of screen events for 2 consecutive days, the first step of the iSenseSleep algorithm is to identify all tuples, that is, consecutive screen events that are separated by at least 4 hours. Second, the iSenseSleep algorithm evaluates the SCREEN ON events in the morning hours after at least 4 hours since the last SCREEN OFF event. The algorithm reasons if the SCREEN OFF event before the SCREEN ON at the wake-up time is really the event when the user went to sleep (along the evening hours) or an event in the middle of the night when he or she woke up and checked the smartphone. For this reason, the algorithm clusters all possible events around this SCREEN OFF event (if there are other events in the range of 5 min) and picks up the previous SCREEN OFF event (before the one in the middle of the night) that is registered at least 2 hours earlier that night. If this is the case, iSenseSleep assumes the last screen event considered for sleep duration calculation as the last one before the sleep break in the middle of the night.

The operation of identifying the possible sleep episodes is repeated until all the screen events (SCREEN OFF and SCREEN ON) are analyzed. Once (1) the function of the algorithm provides the list of time intervals relating to sleep episodes, (2) the evaluation function derives 3 scores to provide a likelihood value for all the episodes to understand which one most likely relates to the overnight sleep episode of the user. To do this, the function (2) assigns a score to the different episodes identified by (1) along 2 consecutive days, comparing it with the assumed *time to bed* (eg, how far it is from 22 hours or 10 pm) and the identified time of wake up, for example, how far it is from a *normal* wake-up time (derived based on the entire smartphone log dataset). Function (2) also assigns a score to a sleep episode by evaluating how much time passed between the usual wake-up time during weekends and weekdays, and what is the total duration of the sleep for this episode. On the basis of these 3 values, the function provides an evaluation of all the possible episodes to select the one that identifies the overnight sleep of the user with the highest probability.

### User Study and Data Collection

For this research, we recruited both young university students (10) and adults (working mothers, 4), 14 in total, to have 2 different groups of individuals and to understand the behavior and the performance of the algorithm with 2 different daily behaviors and daily usage of the smartphone. In particular, students can be considered as more digitally native [23], hence more attached and closer to their phone, while working mothers are probably less attached, as they have an entirely different lifestyle from students and are busy with work and caregiving activities. This is also reflected in the fact that we had aimed to recruit 10 working mothers but given the time constraints on the recruitment and the lack of immediate availability of the potential participants, we failed to do so. The study has been approved by the IRB of University of Copenhagen (Denmark) in 2015 under the protocol number 2015-15-0117/519-0019 /15-5000 and took place at the end of 2015-mid 2016 in Copenhagen, Denmark.

For the women group (S01-S04), we initially recruited 4 working mothers (in their 30s and 40s, caring for at least 1 child), whereas for the student group (S05-S14), we recruited 10 participants (aged between 18 and 30 years, 8 of them are males). For each user, we provided 1 BASIS Peak smartwatch and installed the mQoL-log app on his or her smartphone. The users were encouraged to wear the watch at all times, even for sports and under the shower. The smartphone and smartwatch data were automatically synchronized with the QoL servers without the intervention or interaction of the users.

**Figure 2:**
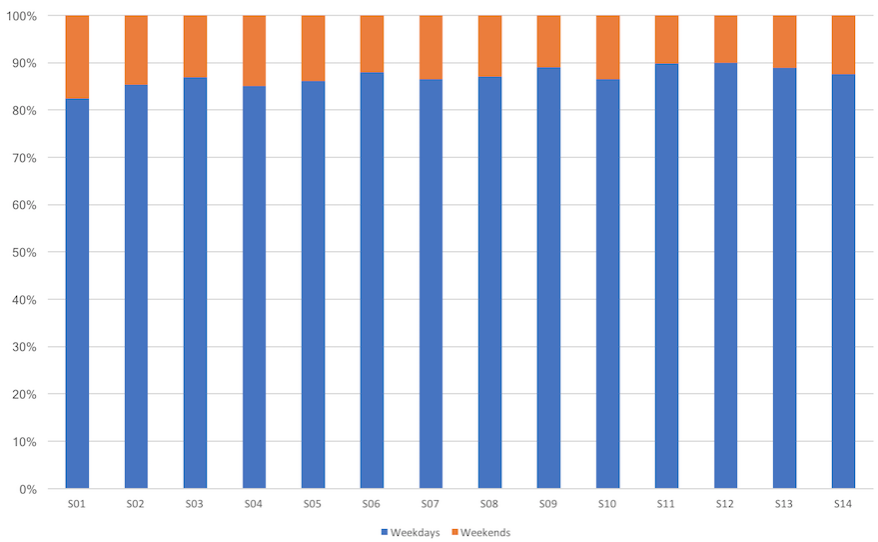
Percentage distribution of week or weekend days for each study subject.

Participation in the study was free, and participants could leave the study whenever they wanted, in addition to having autonomy of wearing the smartwatch or not. The working mothers group provided an average of a bit more than a month of data—that is, 46 days of data (±23), whereas the students’ group—almost 5 months of data—that is, 148 days (±64), with some contributing full 6 months of data. Table 1 provides the total amount of participation days, whereas Figure 2 shows the distribution between weekdays and weekends (Saturday and Sunday).

**Table 1:** Number of participation days (S01-04: working mothers; S05-S14: students).

Subject ID	Weekdays	Weekend days	Total days
S01	14	3	17
S02	70	12	82
S03	40	6	46
S04	34	6	40
S05	187	30	217
S06	168	23	191
S07	147	23	170
S08	167	15	192
S09	145	18	163
S10	115	18	133
S11	114	13	127
S12	45	5	127
S13	16	2	18
S14	190	27	217

### Trial Registration

Study protocol “Understanding Diverse Factors Influencing Individuals’ Sleep Quality And Smartphone-Based Ubiquitous Assessment Of Individual Sleep Patterns”: Protocol 2015-15-0117/519-0019/15-5000 approved by the Institutional Review Board, Faculty of Science, University of Copenhagen, Denmark; Protocol Director: Professor K Wac, Active since 2015.

## Results

### Sleep Duration

The sleep duration derived by the iSenseSleep algorithm presented in the previous section has been compared with the ground truth data provided by the BASIS smartwatch. The average sleep duration, as measured by the BASIS and the iSenseSleep algorithm, are provided for both the working mothers’ group and the students’ group (Table 2).

### Recommended Sleep Duration

**Figure 3:**
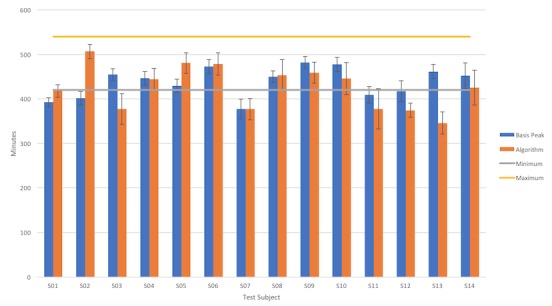
Average sleep duration by the BASIS smartwatch and iSenseSleep versus the recommended sleep per night (gray line: 7 hours=420 min; yellow line: 9 hours=540 min).

Figure 3 provides the data calculated using iSenseSleep algorithm and the BASIS data compared with the recommended amount of sleep hours for healthy adults [24], as we will discuss further in this paper.

We additionally compared differences between the beginning and the end of the sleep, to have a closer look in how much time passes between when the person stops using the smartphone (ie, when the last smartphone event (ON or OFF or PRESENT) is recorded) and he or she falls asleep according to the smartwatch. We report the cumulative average difference between the BASIS smartwatch and iSenseSleep-derived time for the beginning and the end of the sleep. Values are calculated considering the absolute value of the difference. Table 3 shows the average error (SD) of the iSenseSleep algorithm for sleep duration, sleep beginning, and sleep end times for working mothers’ and the students’ group.

As we can see from the results, the error made by the iSenseSleep algorithm is on average approximately 13% of the sleep duration value for the working mothers’ group (corresponding to average of 53 min), or approximately 7% for the students’ group (corresponding to average of 24 min). If we divide the days between weekdays and weekends, the iSenseSleep error is similar for weekdays (12%±10% for working mothers and 7%±6% for students), whereas it increases to 13% when considering only weekends for working mothers and to 9% for the students’ group. Overall, the sleep duration error is persistent for working mothers, while it is lower for weekdays and larger for weekends when considering the student population.

The iSenseSleep error for the start time and the end time of the sleep is slightly higher both for the women and the students’ group. In particular, the starting time error for the working mothers’ datasets is about 108 min (26% of the entire sleep duration), whereas for the students, this error is about 79 min (18%) on average for all days together. During the weekdays, the error is the same for working mothers, and it increases by an additional 18 min for students—resulting in 97 min (23%). The sleep start time error is the same for the working mothers across the whole dataset, whereas it is higher for the students at weekends.

**Table 2:** Average sleep duration comparing the BASIS smartwatch and the iSenseSleep algorithm.

Group and subject ID		Sleep duration (min), mean (SD)					
		All days		Weekdays		Weekend days	
		BASIS	iSenseSleep	BASIS	iSenseSleep	BASIS	iSenseSleep
**Mothers**							
	S01	393 (10)	418 (14)	393 (13)	409 (15)	389 (20)	458 (18)
	S02	402 (15)	507 (16)	406 (17)	509 (25)	378 (23)	493 (19)
	S03	455 (13)	377 (34)	464 (10)	377 (29)	392 (30)	379 (17)
	S04	446 (15)	444 (25)	437 (18)	433 (17)	503 (15)	506 (23)
**Students**							
	S05	429 (15)	481 (23)	436 (15)	483 (20)	386 (29)	465 (15)
	S06	473 (16)	478 (25)	474 (10)	485 (15	461 (22)	429 (24)
	S07	377 (22)	377 (24)	378 (30)	376 (15	374 (23)	384 (37)
	S08	450 (13)	454 (35)	449 (13)	450 (23	453 (35)	479 (17)
	S09	482 (14)	459 (24)	486 (29)	463 (18	443 (35)	429 (27)
	S10	478 (16)	446 (36)	481 (24)	441 (37	453 (27)	476 (19)
	S11	409 (19)	378 (45)	417 (17)	381 (23	344 (34)	350 (24)
	S12	417 (24)	374 (16)	408 (27)	376 (29	501 (24)	362 (16)
	S13	462 (16)	346 (25)	454 (24)	355 (35	519 (12)	272 (23)
	S14	452 (29)	426 (39)	450 (23)	424 (34	468 (29)	434 (26)

**Table 3:** Sleep statistics' differences between the BASIS smartwatch and the iSenseSleep algorithm.

Group		All days, mean (SD)		Weekdays, mean (SD)		Weekend days, mean (SD)	
		Min	Percentage	Min	Percentage	Min	Percentage
**Working mothers**							
	Sleep duration	53 (41)	13 (10)	53 (43)	12 (10)	50 (45)	13 (12)
	Sleep start time difference	108 (28)	28 (8)	108 (32)	26 (9)	108 (43)	27 (12)
	Sleep end time difference	83 (28)	20 (9)	86 (34)	21 (9)	65 (41)	17 (9)
**Students**							
	Sleep duration	24 (17)	7 (4)	32 (27)	7 (6)	41 (40)	9 (8)
	Sleep start time difference	79 (16)	18 (3)	78 (17)	18 (3)	97 (18)	23 (6)
	Sleep end time difference	68 (22)	17 (5)	72 (25)	16 (5)	84 (47)	20 (13)

The end time error is similar to the start time error but higher than the error for the sleep duration. In particular, the working mothers’ group has an average error of 83 min (20%) considering all the days together, or 86 min (21%) for weekdays and 65 min (17%) for weekends. On the other hand, for students, the average error is 68 min (17%) considering all the days together, and 72 min (16%) for weekdays and 84 min (20%) during weekends. The sleep end time error is higher for the weekdays for the working mothers than at the weekends, whereas the opposite is true for the students.

In the following section, we provide a discussion about these results and how they can be interpreted and leveraged when designing technologies helping individuals to develop healthier sleep habits.

## Discussion

### Principal Findings

Results show that based on the smartphone ON-OFF patterns, an individual’s sleep duration can be estimated with an average error of 24 ± 17 min (7% ± 4% of the total duration), enabling estimates of sleep start and wake-up times as well as sleep deprivation patterns.

To evaluate the accuracy of the iSenseSleep algorithm, we calculated the statistical significance between the average duration of the sleep calculated by the algorithm and the ground truth data provided by the BASIS smartwatch. Assuming a normal distribution of the datasets, if the *P* value is larger than .05, it indicates that there is no significant difference between values provided by the two, and therefore, the iSenseSleep algorithm is adequate. We performed a paired, one-tailed *t* test to compare the 2 values of sleep duration. For the all days, for the working mothers’ group, *P*=.35, whereas for the students’ group *P*=.14. We conclude that the iSenseSleep algorithm that is based only on the smartphone interaction data analytics can be used to estimate the sleep duration within a group of subjects. Moreover, *t* test results conducted for each single study subject show the algorithm’s performance within subject. Results are provided in Table 4.

As we can see from Table 4, for less than 50% of participants (6 over 14), the average sleep duration estimated by iSenseSleep is statistically different from the ground truth data; this is mostly because of iSenseSleep underestimating the sleep duration of the individual. Given this result, it is possible to make several conclusions about the performance and validity of our algorithm.

First of all, using only the data related to screen events of the smartphone, for example, SCREEN ON and SCREEN OFF, in this study, we have shown that it is possible to provide an estimate of the smartphone owners’ sleep duration with a small probability of the estimation error being at most around 100 min.

In addition, considering the sleep duration recommendations [24] for the adult category, from the data calculated using the algorithm, it is possible to see that only a few of the users sleep on average enough during the night (eg, S03, S06, S09, and S10), whereas all the others lack sleep. Some of them, for example, S01, S02, S07, and S11, may even suffer from sleep deprivation and should increase the amount of sleep time. For smartphone users who are likely to rely on their phones more, sleep deprivation is very likely to be captured by the iSenseSleep (S01, S07, and S11), despite the sleep duration’s estimation error. For others, such as S03, S12, or S13, the feedback to the user could be to stay away from interacting with their phone during the night (eg, implementing automatic switching OFF function) because even if they are sleeping long enough (as underestimated by the iSenseSleep algorithm), the night phone *sleep breaks* lasting more than 5 min are not healthy.

Moreover, there are other aspects of the algorithm that are interesting. First of all, the accuracy of the algorithm is higher when calculating the duration of the sleep than when evaluating the individual’s sleep start or end time. This result can be explained by the fact that usually when people wake up, before using the phone or turning it ON, they stay in bed without sleeping and just wait for the right moment to get up. Moreover, when going to sleep, even if the smartphone is one of the last things an individual is interacting with, there is an amount of time that passes before the person will effectively fall asleep. These 2 conditions make the identification of the moment of sleep start and wake-up times more challenging than the calculation of the duration of the sleep, which is then shifted in time with respect to sleep start or end time yet adequately estimates the duration.

**Table 4:** Average sleep duration by the BASIS smartwatch versus iSenseSleep algorithm, and statistical significance tests.

Subject ID	Min, mean (SD)		*P* value^b^	Algorithm estimate (Under or over)	Sleep deprived?	
	BASIS	iSenseSleep			BASIS	Algorithm
S01	393 (10)	418 (14)	.31	—^a^	Yes	No
S02	402 (15)	507 (16)	<.001	Over	Yes	No
S03	455 (13)	377 (34)	<.001	Under	No	Yes
S04	446 (15)	444 (25)	.46	—	No	No
S05	429 (15)	481 (23)	<.001	Over	No	No
S06	473 (16)	478 (25)	.27	—	No	No
S07	377 (22)	377 (24)	.50	—	Yes	Yes
S08	450 (13)	454 (35)	.38	—	No	No
S09	482 (14)	459 (24)	.06	—	No	No
S10	478 (16)	446 (36)	.23	—	No	No
S11	409 (19)	378 (45)	.002	Under	Yes	Yes
S12	417 (24)	374 (16)	.20	—	No	Yes
S13	462 (16)	346 (25)	.003	Under	No	Yes
S14	452 (29)	426 (39)	.002	Under	No	No

^a^The iSenseSleep algorithm estimates sleep duration accurately (does not underestimate or overestimate)

^b^Results were deemed statistically significant at *P*<.05: the algorithms differ.

The second aspect we can see from the results is a comparison between weekdays and weekends. Both for the working mothers’ group and the students’ group, the accuracy of the algorithm during weekdays is higher with respect to the accuracy during weekends. This may come from 2 different lifestyle choice aspects. The first one is related to the fact that during the weekends, lifestyle of each person is less *standard* and *coherent* with respect to the rest of the days, such as socializing in the evening, eating together outside of the house, etc, thus strongly reducing the amount of time spent with the smartphone. Interestingly enough, mothers have less error for the wake-up time at weekend, than at the weekdays—meaning that they must pick up their phone early at the weekend to, for example, leave the house with the family. In weekdays, they may be occupied with preparing the children alone—before they pick up their phone when leaving the house. In contrast, the students pick up the phone earlier at the weekdays (exhibiting smaller error for the wake-up times)—most likely being late for school or work, whereas leaving it longer behind at the weekends (most likely staying longer at home before leaving for shopping or weekend activities). On the other hand, the number of weekends is much lower than the number of weekdays in our dataset, thus reducing the accuracy of the algorithm because there is fewer data available to compare with the ground truth.

Comparing the 2 user groups, what is clear is that the accuracy of the algorithm is higher when considering the students’ group, compared with the working mothers’ group. Despite the fact that the number of working mothers is lower than the number of students, what influences the accuracy of the algorithm is probably the users’ lifestyle and the closeness to the smartphone. In particular, students can be considered more digitally native than working mothers, thus making their last interaction with the smartphone closer to their sleep time. Working mothers may have family commitments and make behavioral choices resulting in a different relation between their sleep and their smartphone usage.

### Limitations

iSenseSleep provides an estimation of the sleep duration patterns of its user; however, it has some study limitations as well as algorithm-specific limitations that can reduce its total accuracy.

Overall, the limitation of the algorithm is that it may not be representative for all populations and be more suitable for *digitally native* populations [23]. Overall, given the small sample of working mothers, the results gathered for this population are rather indicative and cannot be conclusive. We admit that limitation and at the same time indicate that, overall, as mobile users become increasingly more attached to their phones, the algorithm will be able to provide more accurate sleep assessments for a larger variety of populations than initially planned for, for example, for older users relying on smartphone for their daily life tasks, including using their smartphone as an alarm clock [15].

Somehow related to this, a limitation of the algorithm may stem from the choice of baseline method for its comparison. Namely, leaving out a choice of the smartwatch datasets as a baseline, we could have selected other, more smartphone-related baseline methods. In the related work section, we indicate the other smartphone or HCI-analysis methods with an error of 42 min (Chen et al [25]) or 45 min (Abdullah et al [26]) for sleep duration; these methods leveraged microphone and luminosity sensors besides, for example, charging patterns. In contrast, in this work, we leverage only ON or OFF button of the smartphone without privacy-sensitive data sources such as microphone. In addition, our results are supported by 14 participants engaged in data collection of up to 6 months compared with 8 people 1 week per person study (Chen et al) or a study with 9 persons for 3 months (Abdullah et al). Therefore, even if the choice of baseline may seem to be a limitation, we claim that the choice of validated BASIS smartwatch data is motivated toward this end.

As the first limitation related to the algorithm itself, we recognize that the algorithm is suitable for modeling sleep duration and sleep patterns of users who study or work in conventional daylight hours [27], and it is not suitable for modeling sleep duration and sleep patterns of users who study or work on shifts or along unconventional hours (7% of population of employed adults in Europe and 16% in the United States [27]), who do not sleep during *standard* sleeping times but whenever they can depending on working shifts. This problem is not easy to solve using only the screen events of the smartphone, as the algorithm assumes sleeping episodes occurring during the night (after 10 pm or 22 hours), thus penalizing the possible sleeping episodes that are not within this time range. On the other hand, removing the penalization of the possible sleep episodes that are far from the night-sleep times imposes to calculate the likelihood function using only, for example, the length of the possible sleep duration, hence strongly reducing the accuracy of the algorithm in standard conditions and sleeping patterns. For example, imagine a primary school teacher not touching the phone over consecutive 6 or 8 hours of work time, which would end up being assessed as sleeping along the day, while completely inaccurate. One possible way to overcome this limitation is to introduce a short user profile that may calibrate the iSenseSleep given the individual’s self-declared *standard* sleeping times.

The second limitation is related to the usage of the smartphone by the user and is something that is already highlighted by the accuracy of the algorithm during the weekends or with the working mothers’ group. In particular, if the individual is not very attached to the smartphone, meaning that the smartphone is not used frequently along the day, especially in the evenings, the algorithm overestimates the time the user goes to bed and underestimates the time when he or she wakes up, as there is a non-negligible delay between the last and the first smartphone interaction versus when the user goes to bed and wakes up. To mitigate this problem, one possible solution is to use other sensors available on the smartphone, such as, for example, the accelerometer and built-in activity recognition, or sense if the phone is charging, to understand if the smartphone, and therefore the user, is potentially still awake and moving around.

### Comparison With Previous Work

This paper aims to understand in which way inclusion of a smartphone in our everyday life activities can be leveraged to understand our sleep patterns. For example, Dey et al [28] conducted an empirical experiment estimating how close the smartphone is to its user along daily life activities. With this study, they showed that the smartphone is at the room distance for almost 90% of the time and that it is possible to predict the proximity level of the smartphone with about 80% accuracy with features simple to collect and model on the smartphone itself (eg, Wi-Fi AP name). This and studies by Patel et al [29,30] show how smartphones are getting closer to their users day by day; hence, they can be leveraged to longitudinally and in a minimally obtrusive and lightweight way sense users’ lifestyle and infer some aspects (including sleep) without users’ intervention or explicit data input.

As sleep is one of the most important aspects of our life, several researchers have focused their attention on how it is possible to understand and infer sleep patterns of smartphone users. For example, Abdullah et al [26] showed how phone usage patterns could be used to detect and predict individual daily variations indicative of temporal preference, sleep duration, and sleep deprivation. They followed 9 participants for 97 days and collected ground truth data using a manual journal where participants annotated their sleep moments during the night. An average sleep duration error among all the participants was about 45 min (10% of the average sleep duration).

In addition, Lane et al [31] assessed the sleep, physical activity, and social interactions leveraging several smartphone sensors; for sleep, they leverage phone charging patterns, accelerometer, as well as microphone data to understand the noise level of the environment the user is in and to classify the activity as sleeping (or not). Lane et al used logistic regression models for sleep duration estimate, and the results were provided only for 5 persons, each collecting 1-week of data via self-reports. The error with respect to the sleep duration was reaching up to 1.5 hours.

Chen et al [25] developed an algorithm for sleep detection using sensors available on the smartphone, in particular, charge events, time, and length of smartphone usage and microphone or luminosity sensor. Combining all the information available, they developed the Best Effort Sleep model, which was tested on a 1-week 8-person study comparing the calculated data with the Zeo headband and the Jawbone wristband, resulting in an average error of sleep duration of about 42 min.

Contrary to the above-mentioned works of Abdullah et al [26], Lane et al [31], and Chen et al [25], iSenseSleep approach results in smaller average error for the sleep duration without using privacy-sensitive datasets from the user’s smartphone. Furthermore, we have used wearable datasets as a ground truth, to avoid the use of subjective self-reported data used by Abdullah et al [26].

Min et al [32] developed an algorithm for sleep detection based on smartphone sensors, and in particular, using the accelerometer, the microphone, the ambient light sensor, the screen proximity sensor, the running processes, the battery state, and the display screen state. They used all these sensors to develop an algorithm tested with 27 participants during 1 month. Ground truth data were collected using a sleep diary. The system classified correctly sleep state with a 93% accuracy and overall sleep quality with an 81% accuracy. Sleep duration was neither modeled nor evaluated.

Hao et al [33] developed iSleep, a smartphone app that uses the built-in microphone of the smartphone to detect the events that are related to sleep quality, such as body movements, coughing, and snoring, and infer quantitative measures of sleep quality. The model is based on a decision-tree algorithm to classify various events and calculate acoustic features. They tested the algorithm with 7 participants during 51 nights of sleep, and the accuracy was above 90% for event classification in different scenarios. Sleep duration was neither modeled nor evaluated. Contrary to Min et al [32] and Hao et al [33], iSenseSleep approach focuses exclusively on the sleep duration without leveraging the privacy-sensitive datasets from the user’s smartphone.

Furthermore, the authors of Somnometer (by Shirazi et al [34]) developed their own app (also connected to social network) that acts as an alarm clock, and they have evaluated the sleep duration for an individual based on data collected within the app. The authors compared the sleep duration assessed by the app with the wearable device (HedgeHog) for 20 nights and concluded that their app can be used as a sleep duration sensor (without providing numerical results supporting its accuracy). The work on Somnometer focused then on the social sharing features and their update among the Somnometer users.

Compared with the presented approaches, our approach is slightly different because it aims at understanding sleep duration of each individual considering only the interaction they have with their phone, without using information collected from other sensors, especially privacy-sensitive sensors such as microphone, used by many other authors indicated above. Moreover, ground truth data are not collected using diaries or other methods that are affected by a subjective self-report error but using objective data collected from a smartwatch. In this research, we show how smartphones are becoming accurate proxies of our everyday life and that they can be easily used to provide an estimation of individuals’ sleep duration.

### Conclusions

Smartphones are getting becoming increasingly ubiquitous every day, getting closer to their owners, being carried around in a pocket, and becoming more integrated into the everyday life of individuals. This proximity of the smartphone with the daily life of users opens the door to many different opportunities for leveraging smartphone use to bring more understanding of the daily activities and routines of the users.

In this study, we presented the possibility of evaluating users’ sleep patterns by analyzing their interaction with their smartphone, and in particular, only the smartphone screen interaction data (screen ON and OFF). The approach presented here, denoted iSenseSleep; is lightweight; nonintrusive, as it does not affect individuals’ life; privacy preserving because it does not use any privacy-related information (eg, phone microphone or light sensors); and it is low cost because it does not require any other external devices for sleep duration estimation. iSenseSleep has been evaluated against the wearable BASIS peak datasets for 2 different user groups, one consisting of 4 working mothers, and another comprising 10 students. These 2 groups can be considered very different from each other because the second one is significantly more digitally native than the first one; usually the usage of smartphone is higher in adolescents and young adults than in adults. Results show how, on average, the difference between the sleep duration calculated with the algorithm and the ground truth data of the smartwatch is about 53 min for the working mothers’ group and 24 min for the students’ group, that is about 13% and 7% with respect to their total sleep duration. This error is almost the same for weekdays and slightly higher (13% and 12%) for weekends. Moreover, the results show that the difference in sleep duration evaluated by the iSenseSleep and by the BASIS is not statistically significant. These results support the possibility of using smartphones as a nonintrusive, cheap sleep duration pattern analyzer.

In the future, we plan to increase the accuracy of the algorithm to have more precise data about individuals’ sleep behavior (beyond the sleep duration), relying on, for example, historical trends of the user’s behavior that could help us understand, which are the sleep episodes, and to analyze if and to what extent, there is a correlation between sleeping patterns of the users and their interaction with the smartphone in terms of usage time, applications used, etc. In addition, more specific efforts will be provided for estimating the wake-up time—as a consistent wake-up time is being recognized by the medical experts as an important contributor to one’s wellness and health state in the long term.

## Abbreviations

mQoL-log: mobile quality of life logger
QoL: Quality Of Life
